# Suggested organism entry portal of necrotizing fasciitis with complete DNA from fascia, blood, and pharyngeal ulcers: A case report

**DOI:** 10.1016/j.amsu.2022.104732

**Published:** 2022-09-30

**Authors:** Satoru Murata, Chie Toyoshima, Satoshi Suzuki, Norio Sato

**Affiliations:** a Department of Emergency Medicine and Critical Care Medicine, Ehime University Graduate School of Medicine, Japan; b Department of Emergency Medicine and Intensive Care Medicine, Saiseikai Noe Hospital, Japan

**Keywords:** Necrotizing soft tissue infection, Pulsed field gel electrophoresis, Group A streptococcus, MRI, Magnetic Resonance Imaging, CT, Computed Tomography, ICU, Intensive Care Unit, LRINEC, Laboratory Risk Indicator for Necrotizing Fasciitis, CHDF, Continuous hemodiafiltration

## Abstract

**Introduction and importance:**

In approximately 50% of patients with necrotizing fasciitis, infection begins deep in the soft tissues. A history of trauma is often absent. The mechanism of spread has not been elucidated. We report a case of type II necrotizing fasciitis in which the streptococcal strain was identical to isolates from other locations in the same patient.

**Case presentation:**

A 42-year-old man presented with left leg pain. Two days prior, he had a non-penetrating injury to the left thigh while playing futsal. Workup revealed swelling of the left gastrocnemius. He was admitted to orthopaedics. On the third hospital day, he was referred to our department for hypotension, impending respiratory failure, and decreased sensorium, and subsequently admitted to the ICU. A biopsy was done on the left gastrocnemius fascia. He was diagnosed with necrotizing fasciitis. On the seventh hospital day, left hip amputation and extensive debridement of the trunk were done. Patient improved and eventually recovered.

**Clinical discussion:**

Group A streptococcus was isolated in from the fascia, blood, and pharyngeal ulcer. Pulsed field gel electrophoresis showed all isolates to be genetically identical. An oral route of infection was considered.

**Conclusions:**

This is the first report in which etiologic agent of necrotizing fasciitis is genetically identical with isolates from other parts in the absence of trauma.

## Highlights

• Point of entry for bacteria occurs after a non-penetrating injury is unknown.
• The same strain from the fascia was isolated from blood, and a pharyngeal ulcer.
• This is the first report of entry portal for necrotizing fasciitis via the mouth.

## 1 Introduction and importance

Necrotizing soft tissue infection is a disease in which necrosis can rapidly spread. When the fascia is involved, the condition is called necrotizing fasciitis, which carries a high mortality rate. Type II necrotizing fasciitis is caused by group A streptococci. However, its mode of entry is yet to be elucidated [1], leading to delayed diagnosis and treatment.

We present a case of necrotizing fasciitis from a bruise on the lower leg. Cultures from the affected fascia, blood, and an oral ulcer isolated genetically identical streptococci.

This study complies with the SCARE guidelines. The patient provided informed consent for publication. We declare no competing or conflict of interest [2].

## 2 Case presentation

A 42-year-old man presented to our institution with left leg pain. His past medical and social history were unremarkable and he was not on any medicine. Two days prior, he sustained a bruise on his left lower leg after playing futsal. He had no other symptoms. The following day, he complained of pain in that area, leading to a consult and this admission.

On admission, compartment syndrome was suspected; he was then admitted to orthopaedics. His left gastrocnemius muscle was swollen and painful, and MRI showed inflammation and swelling of his gastrocnemius muscle. Orthopaedic surgeons judged that the muscle was injured and admitted the patient for follow-up.

**Fig. 1:**
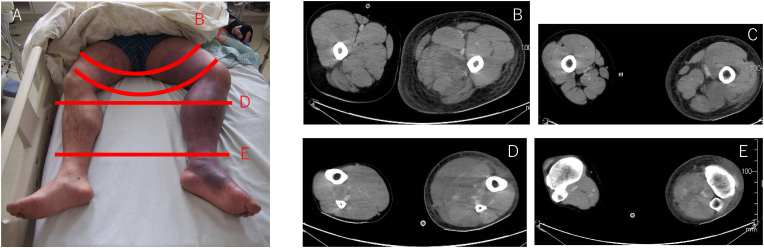
**Physical findings**. Panel A shows the patient's lower extremities. Erythema and swelling were noted on the left upper and lower extremities. Panels B–E show the limbs on the third hospital day. Subcutaneous and muscle swelling were observed.

On the third hospital day, he had exertional dyspnoea on ambulation, progressing to severe respiratory distress. His oxygen saturation was approximately 80%; pulse rate, 130 beats per minute; and blood pressure, 85/45 mmHg. Table 1 shows his arterial blood gas analysis results. Pulmonary thromboembolism had been initially considered; however, computed tomography (CT) was negative. On repeat thorough physical exam, purpura and epidermal swelling were noted on his left thigh (Fig. 1). He was transferred to the ICU for circulatory and ventilatory support, and continuous hemodiafiltration. Table 2 shows his laboratory results on the 1st and 3rd hospital days.

**Table 1:** Arterial blood gas on the third hospital day.

ABG	day 3
FiO2	0.5
pH	7.321
pCO2	20.7 mmHg
pO2	150 mmHg
SBE	−14.6 mmol/L
HCO3	10.4 mmol/L
Lac	6.5 mmol/L

**Table 2:** Laboratory examination results on the first (admission) and third (first ICU day) hospital days with LRINEC score.

	day 1	day 3
WBC	12200	7200/ul
RBC	497	504 × 10^4/ul
Hb	15.1	15.1 g/dl
Ht	41.9	41.2 %
Plt	14.7	4 × 10^4/ul

PT	13.3	15.4 sec
PT-INR	1.18	1.36
APTT	37.1	62.8 sec
D-Dymer		13 ug/mL

TP	6.7	3.9 g/dl
Alb	3.2	1.2 g/dl
Glu	159	94 mg/dl
BUN	33.6	61.8 mg/dl
Cre	1.4	2.6 mg/dl
Na	131	129 mEq/l
K	4.2	5.5 mEq/l
Cl	94	102 mEq/l
AST	42	735 IU/l
ALT	29	168 IU/l
LDH	303	710 IU/l
CK	493	53579 IU/l
T-Bil	1.5	0.4 mg/dl
CRP	42.11	37.01 mg/dl

LRINEC score	6	8

LRINEC, Laboratory Risk Indicator for Necrotizing Fasciitis; ICU, intensive care unit.

Fascial biopsy and Gram staining demonstrated Gram-positive cocci. The patient was diagnosed with necrotizing fasciitis. Antibiotics were started as follows: piperacillin sodium 12 g/day and clindamycin phosphate 2.7 g/day. He also received immunoglobin 10 g/day. However, surgical management was postponed due to poor surgical tolerance.

On improvement, left hip transection and trunk debridement were done on the 7th hospital day. He was extubated on the 11th hospital day and withdrawn from CHDF on the 24th hospital day. Additional debridement and skin grafting were performed. The patient continued to improve clinically until he was discharged on the 157th hospital day. Follow-up is still ongoing.

### 2.1 Biological examination

**Fig. 2:**
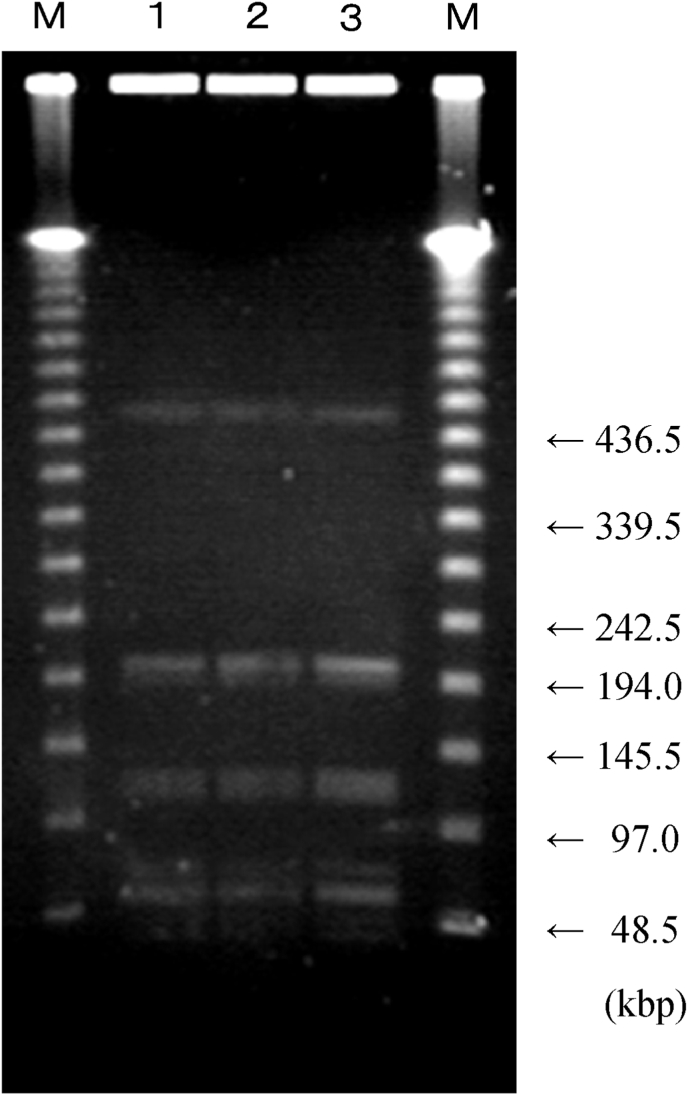
**DNA fragment electrophoresis.** DNA extracted from the bacterial isolates from the fascia, blood, and pharyngeal ulcer are genetically identical. M: DNA size marker (Lambda Ladder), 1: Left Gastrocnemius Fascia, 2: Blood, 3: Pharyngeal Ulcer.

Group A *Streptococcus* was detected in all of the specimens collected on the 3rd hospital day: left gastrocnemius fascia, blood, and pharyngeal ulcer. DNA was extracted from all cultures, cleaved with Sam I, and analysed with the pulse field (SRL, Inc.) electrophoresis (Fig. 2). All bacterial isolates from the three specimens were genetically identical.

## 3 Clinical discussion

To date, there is no universal and accurate method to establish bacterial mode of entry and spread in necrotizing fasciitis. We could not identify the route in this case. Moreover, the same strain was isolated from the fascia, blood, and a pharyngeal ulcer. No data in literature support or similarly report on a monobacterial multifocal infection with a necrotizing fasciitis component. According to the literature, the portal of entry is not implicated in the mode of spread in type II necrotizing fasciitis [1,3,4]. However, a hypothesis is that an asymptomatic pharyngeal carriage of *Streptococcus pyogenes* can cause transient bacteraemia, leading to fascial seeding via attachment to damaged fascial vimentin [5,6]. We believe that our report will greatly contribute to the investigation of the cause and invasion route of the etiologic agents in type II necrotizing fasciitis.

Necrotizing soft tissue infection is one of the most lethal bacterial infections. When fascia is involved, it is called necrotizing fasciitis. It is classified into Types I to III according to the causative organism and patient background [[6], [7], [8], [9], [10]]. Type I is a polymicrobial infection and is usually seen in elderly or immunocompromised patients. This type is often associated with gas formation in the tissues. Hence, other soft tissue infections must be distinguished and ruled out. Necrotizing fasciitis type II is a monomicrobial infection most commonly caused by group A streptococci, followed by *Staphylococcus aureus,* that often affects the general population [11,12]. Type III involves minor penetrating trauma and is caused by aquatic bacteria such as *Aeromonas hydrophila* and *Vibrio vulnificus* [13].

The clinical manifestations of necrotizing fasciitis range from local soft tissue oedema, erythema, severe pain, tenderness, and fever to skin bullae, necrosis, and sepsis. The Laboratory Risk Indicator for Necrotizing Fasciitis (LRINEC) score [14], used to aid in diagnosis, comprises total white cell count and haemoglobin, sodium, glucose, creatinine, and C-reactive protein levels [15]. It has a reported sensitivity of 92.0% and a specificity of 96.0%. Necrotizing fasciitis becomes more likely with a score of 6 or higher.

The patient scored 6 points on the first hospital day and 8 points on the 3rd hospital day. Further workup strengthened the suspicion of necrotizing fasciitis (Table 2). Necrotizing fasciitis progresses very rapidly, thereby necessitating timely and appropriate diagnosis and treatment. Delay increases the risk of amputation or even death. There are 100–200 cases of fulminant streptococcal infection reported annually in Japan, with a mortality rate as high as 30% [16]. The timing of surgical intervention and adequate resection is extremely important. Delay can expedite deterioration, increase morbidity and mortality, and delay recovery.

We hypothesize that the pathogenesis in this case has been influenced by the initial deep, nonpenetrating tissue injury that stimulated the repair response, leading to an influx of leukocytes and activation and proliferation of myogenic progenitor cells [6]. Second, in the setting of antecedent transient bacteraemia, microorganisms may be directed to the foci of injury and repair. During the process of healing, increased vimentin expression on activated myogenic progenitor cells and infiltrated macrophages serves as a ligand for group A *Streptococcus* receptors. Third, bacterial proliferation with consequent local production of exotoxins, such as streptolysin O and streptococcal pyrogenic exotoxin A, may activate platelets. This results to a localized intravascular microthrombosis and vascular occlusion in postcapillary venules, arterioles, and even larger vessels. Finally, vascular occlusion leads to ischemic injury to surrounding tissue.

Cutaneous manifestations on necrotizing fasciitis, such as ecchymoses and bullae, develop in the late phase of infection. Group A *Streptococcus* is part of the normal oropharyngeal flora [17]. However, it was difficult to attribute this case to oral resident flora because it could not be shown that onset of ulceration in the oral cavity occurred prior to bacteriology workup.

The findings in this case may help link preceding infections or bloodstream infections by colonizing bacteria to newer infections or foci. We recommend paying attention to seemingly unrelated lesions including oral ulcers and to microorganisms that are often considered seemingly harmless.

## 4 Conclusion

We described a case of necrotizing fasciitis originating from the left lower leg. Genetically identical group A *Streptococcus* was detected in all fascia, blood, and pharyngeal ulcers. We believe that a preceding pharyngeal ulceration may be a potential portal of entry for organisms that ultimately cause necrotizing fasciitis. We recommend observing future cases to clarify whether the preceding pharyngeal ulceration may be a gateway to bacterial invasion as a cause of necrotizing fasciitis.

## Ethical approval

This study was approved by the Ethics Committee of institution.

## Sources of funding

The authors received no funding or financial support for this work.

## Author contribution

Satoru Murata: writing the paper, treatment of this patient, and collect data.

Chie Toyoshima: advice for the paper, treatment this patient, and collect data.

Satoshi Suzuki: advice for the paper, treatment this patient, and collect data.

Norio Sato: advice for the paper.

## Research registration number

Our paper does not include new methods or techniques. This report only includes the new observations.

## Guarantor

Satoru Murata, Ehime University Graduate school of Medicine, Department of Emergency and Critical Care Medicine, Address: 454 Shitsukawa, Toon City, Ehime Prefecture, 791-0295, JAPAN, TEL: +81-89-960-5722, FAX: +81-89-960-5714, E-mail: smurata@kuhp.kyoto-u.ac.jp.

## Consent

The patient has provided written informed consent for participation and publication of this study.

## Informed consent statement

Written informed consent was obtained from the patient for publication of this case report and accompanying images. A copy of the written consent is available for review by the Editor-in-Chief of this journal on request.

## SCARE guidelines

The authors have read SCARE guidelines and have complied with its provisions.

## Provenance and peer review

Not commissioned, externally peer-reviewed.

## Data availability statement

Not applicable.

## Declaration of competing interest

The authors declare no competing or conflict of interest.
